# Alagille Syndrome Candidates for Liver Transplantation: Differentiation from End-Stage Biliary Atresia Using Preoperative CT

**DOI:** 10.1371/journal.pone.0149681

**Published:** 2016-02-22

**Authors:** Sook Min Hwang, Tae Yeon Jeon, So-Young Yoo, Ji Hye Kim, Ben Kang, Yon Ho Choe, Haeyon Cho, Jung Sun Kim

**Affiliations:** 1 Department of Radiology and Center for Imaging Science, Samsung Medical Center, Sungkyunkwan University School of Medicine, Seoul, 135–710, Korea; 2 Department of Pediatrics, Samsung Medical Center, Sungkyunkwan University School of Medicine, Seoul, 135–710, Korea; 3 Department of Pathology, Samsung Medical Center, Sungkyunkwan University School of Medicine, Seoul, 135–710, Korea; Texas A&M Health Science Center, UNITED STATES

## Abstract

**Purpose:**

To compare preoperative CT findings before liver transplantation between patients with Alagille syndrome (AGS) and those with end-stage biliary atresia (BA).

**Materials and Methods:**

The institutional review board approved this retrospective study. Eleven children with AGS (median age, 19.0 ± 13.0 months; male to female ratio, 3:8) and 109 children with end-stage BA (median age, 17.9 ± 25.8 months; male to female ratio, 37:72) who underwent abdomen CT as candidates for liver transplant were included. CT images were reviewed focusing on hepatic parenchymal changes, vascular changes, presence of focal lesions, and signs of portal hypertension.

**Results:**

Hepatic parenchymal changes were present in 27% (3/11) of AGS patients and 100% (109/109) of end-stage BA patients (*P* < .001). The hepatic artery diameter was significantly smaller (1.9 mm versus 3.6 mm, *P* = 008), whereas portal vein diameter was larger (6.8 mm versus 5.0 mm, *P* < .001) in patients with AGS compared with patients with end-stage BA. No focal lesion was seen in patients with AGS, whereas 44% (48/109) of patients with end-stage BA had intrahepatic biliary cysts (39%, 43/109) and hepatic tumors (8%, 9/109) (*P* = .008). Splenomegaly was commonly seen in both groups (*P* = .082), and ascites (9% [1/11] versus 50% [54/109], *P* = .010) and gastroesophageal varix (0% [0/11] versus 80% [87/109], *P* < .001) were less common in patients with AGS than in patients with end-stage BA.

**Conclusion:**

Fibrotic or cirrhotic changes of the liver, presence of focal lesions, and relevant portal hypertension were less common in patients with AGS than in patients with end-stage BA.

## Introduction

Alagille syndrome (AGS) is an autosomal dominant multisystem disorder that may affect the liver, heart, bone, face, eyes, and other organs with variable penetrance [1]. AGS is caused by mutations in the genes encoding *JAG1* or the *NOTCH2* receptor [2, 3]. Liver involvement ranges from asymptomatic biochemical abnormalities to end-stage liver disease [4]. Among patients with severe cholestasis in infancy or early childhood, an estimated 20–30% of patients with AGS will require liver transplantation before they reach adulthood [5, 6]. Survival rates of AGS patients with no liver transplant are 51% and 38% at 10 and 20 years, respectively [7].

The most common leading cause of pediatric liver transplantation is biliary atresia (BA). BA is a destructive inflammatory obliterative cholangiopathy that affects both the intrahepatic and extrahepatic bile ducts. If untreated, BA progresses to cirrhosis and hepatic failure leading to death within the first 2 years of life [8].

AGS and BA, both commonly recognized cholestatic liver diseases in the pediatric population, can develop jaundice at similar ages of neonates and infants and progress to biliary cirrhosis. On histopathologic analyses, AGS is characterized by paucity of interlobular bile duct (PIBD) with development of pericellular fibrosis, whereas distinctive features of BA are the generation of vigorous ductular proliferation with portal fibrosis [9]. We hypothesized that this histopathologic difference may affect imaging findings, and the clinical course of these two cholestatic cholangiopathies. Therefore, the aim of our study is to compare the preoperative CT findings before liver transplantation between patients with AGS and those with end-stage BA.

## Materials and Methods

### Patients

This retrospective study was approved by Samsung Medical Center institutional review board, and the requirement for informed consent was waived. And patient information was anonymized and de-identified prior to analysis. Cases were defined as patients with AGS undergoing liver transplantation, whereas controls were patients with end-stage BA who were candidates for liver transplant. Inclusion criteria mandated that all patients were younger than 18 years of age and underwent contrast-enhanced abdomen CT for pre-operative evaluation.

The records of children who were admitted to our institution as potential candidates for liver transplant between September 2000 and March 2015 were retrospectively searched, yielding 12 patients with AGS and 124 patients with end-stage BA. Among 12 patients with AGS, one patient did not undergo pre-operative contrast-enhanced CT. Among 124 patients with end-stage BA, 15 patients were excluded because of no pre-operative contrast-enhanced CT (n = 10), candidate for re-liver transplantation (n = 2), and no available CT images (n = 3). Finally, 120 consecutive children with AGS (n = 11, 3 boys and 8 girls) and end-stage BA (n = 109, 37 boys and 72 girls) were selected. The charts were reviewed for demographic information, clinical profiles, and laboratory data.

Recent diagnosis of AGS can be made when three or more of the following clinical features are present: chronic cholestasis, cardiac disease, skeletal anomalies, ocular abnormalities, or peculiar faces [10, 11]. In our study, all 11 patients met recent diagnostic criteria for AGS. Liver disease in AGS was diagnosed based on clinically profound cholestasis (n = 11) and histopathologic analysis of either liver biopsy specimens (n = 4) or explanted liver (n = 9). The diagnosis of BA was confirmed by surgical cholangiography (n = 15) and histopathologic analysis of either bile remnants (n = 25) or explanted liver (n = 109).

### Clinical profiles and laboratory data

The charts were reviewed for demographic information, clinical profiles, and laboratory data. The electronic medical records were reviewed, focusing on the patients’ demographics and frequencies of major clinical features of AGS. Biochemical hepatic function tests including aspartate aminotransferase (AST), alanine aminotransferase (ALT), and bilirubin level before liver transplantation were also evaluated. AST:platelet ratio index was calculated as the ratio of (AST/upper limit of normal value: 45 IU/l)/platelet counts (10^9^/l) x 100 [12].

### Image acquisition

All CT examinations were performed using multidetector CT machines (LightSpeed 16 or LightSpeed VCT XT, GE Medical Systems, Milwaukee, WI, USA) with a low dose technique based on patient’s weight and automatic exposure control. Three-phase (arterial, portal, and venous phase, n = 7), two-phase (arterial and portal-venous phase, n = 2), or single portal phase (n = 111) were obtained after injection of intravenous contrast material (2 ml/kg [maximum 120 ml] iomeprol, 300 mg iodine/ml; Iomeron 300, Bracco, Milan, Italy). Imaging parameters were 80–120 kV and 50–150 mA with a 2.5–5 mm section thickness. The mean interval between CT examination and liver transplant was 1.6 months (range 4 days -14 months; median 2 months).

### Image analysis

Two pediatric radiologists (with 13 and 6 years of experience of pediatric imaging interpretation, respectively) retrospectively reviewed all images in consensus. Although the reviewers knew that all patients were candidates for liver transplant, they were blinded to the clinical and histopathologic information.

The CT images were evaluated with an emphasis on hepatic parenchymal changes, presence of focal lesions, vascular changes, and signs of portal hypertension. Hepatic parenchymal changes were classified as heterogeneous parenchymal enhancement, surface nodularity, fissure widening, lobe hypertrophy, and periportal edema. Focal lesions were divided into intrahepatic biliary cysts and hepatic tumors. For evaluation of vascular changes, diameters of the portal vein and hepatic artery were measured at the level of right proximal hepatic artery running parallel to the right portal vein. Measurement of the diameters was taken twice at a workstation using electronic calipers, and the results were then averaged. The ratio of the diameter of the right hepatic artery to the diameter of the right portal vein was obtained. The signs of portal hypertension were determined by splenomegaly, ascites, or gastroesophageal varix. The measurement of spleen size was the maximal dimension at the level of hilum. Splenomegaly was defined according to patients’ age [13].

### Statistical analysis

Statistical analyses were performed using SPSS software (SPSS, version 23; SPSS, Chicago, IL, USA). Descriptive data were summarized as the mean ± standard deviation (SD) for continuous variables and as frequencies for categorical variables. Statistical differences were compared using either two-sample t-test or Mann-Whitney test for continuous variables and Fisher’s exact test for categorical variables. A *P*-value less than 0.05 was considered to indicate a statistically significant difference.

## Results

### Clinical profiles and laboratory data

The demographic information and laboratory data for patients with AGS and end-stage BA are summarized in Table 1. There was a female predominance in both groups. Nine patients (82%, of 11) with AGS and 109 patients (100%, of 109) with end-stage BA underwent liver transplantation; the remaining 2 patients with AGS were placed on the liver transplant waiting lists. Four patients with AGS underwent Kasai portoenterostomy at a mean age of 63.3 days (two at outside institution and two at our institution) because AGS had been misdiagnosed as BA in early infancy. Biochemical hepatic function tests before liver transplantation showed no difference between the groups. The frequencies of major clinical features and results of gene analyses of AGS are presented in Table 2. Nine patients (82%, of 11) underwent molecular confirmation of AGS and all nine had *JAG1* gene mutation. All patients with AGS and BA presented with cholestatic jaundice. The indications for liver transplantation in AGS patients were intractable pruritus (n = 10), failure to thrive (n = 9), severe hypercholesterolemia (> 500 mg/dL, n = 7), osteodystrophy (n = 2), or synthetic liver failure (n = 2). Patients with end-stage BA underwent liver transplantation because of synthetic liver failure (n = 89), uncontrolled portal hypertension (n = 62), refractory cholestasis (n = 52), recurrent cholangitis (n = 35), or failure to thrive (n = 7).

**Table 1:** Clinical profile and laboratory data.

Variables	Alagille syndrome (n = 11)	End-stage biliary atresia (n = 109)	*P*-value
Male: female	3:8	37:72	0.749
Age at CT scan (months)^a^	19.0 ± 13.0	17.9 ± 25.8	0.425
Age at liver transplantation (months)^a^	24.6 ± 31.3	19.5 ± 26.1	0.948
Previous Kasai procedure^b^	4/11 (36%)	100/109 (92%)	0.001
Total bilirubin	18.8 ± 14.9	14.7 ± 9.2	0.388
Direct bilirubin	14.6 ± 14.5	10.2 ± 6.4	0.351
AST	303.3 ± 199.7	272.3 ± 229.4	0.666
ALT	249.1 ± 139.5	177.9 ± 189.2	0.227
AST: platelet	10.3 ± 27.7	5.1 ± 8.9	0.284

Note.–Unless otherwise noted, data are means ± standard deviation. AST = aspartate aminotransferase; ALT = alanine aminotransferase.

^a^Data are median ± standard deviation.

^b^Data are numbers of patients with percentages in parentheses.

**Table 2:** Major clinical features and gene analyses in 11 patients with Alagille syndrome candidates for liver transplantation.

No.	Sex/Age	Liver	Heart	Bone	Eyes	Face	*JAG1* mutation
1	F/4y	PIBD	PS	Butterfly vertebra	NE	Present	NE
2	F/3y	PIBD	PS	Normal spine	NE	Present	NE
3	F/10m	PIBD	PS	Butterfly vertebra	Iris abnormality	Present	Present
4	F/16m	PIBD	PA hypoplasia	Butterfly vertebra	Normal	Present	Present
5	M/22m	PIBD	PS, ASD	Normal spine	Normal	Present	Present
6	F/6m	PIBD	VSD	Butterfly vertebra	NE	Present	Present
7	M/6m	PIBD	PS	Normal spine	NE	Present	Present
8	F/7m	PIBD	PS, ASD	Butterfly vertebra	NE	Normal	Present
9	F/16m	PIBD	PS, ASD	Normal spine	Retinitis pigmentosa	Present	Present
10	F/4m	PIBD	PS	Normal spine	Posterior embryotoxon	Present	Present
11	M/10y	PIBD	PS	Butterfly vertebra	Normal	Present	Present

Note.–y = years; m = months; PIBD = paucity of interlobular bile duct; PS = pulmonary artery stenosis; PA = pulmonary artery; VSD = ventricular septal defect; ASD = atrial septal defect; NE = not evaluated.

### Imaging findings

**Fig 1:**
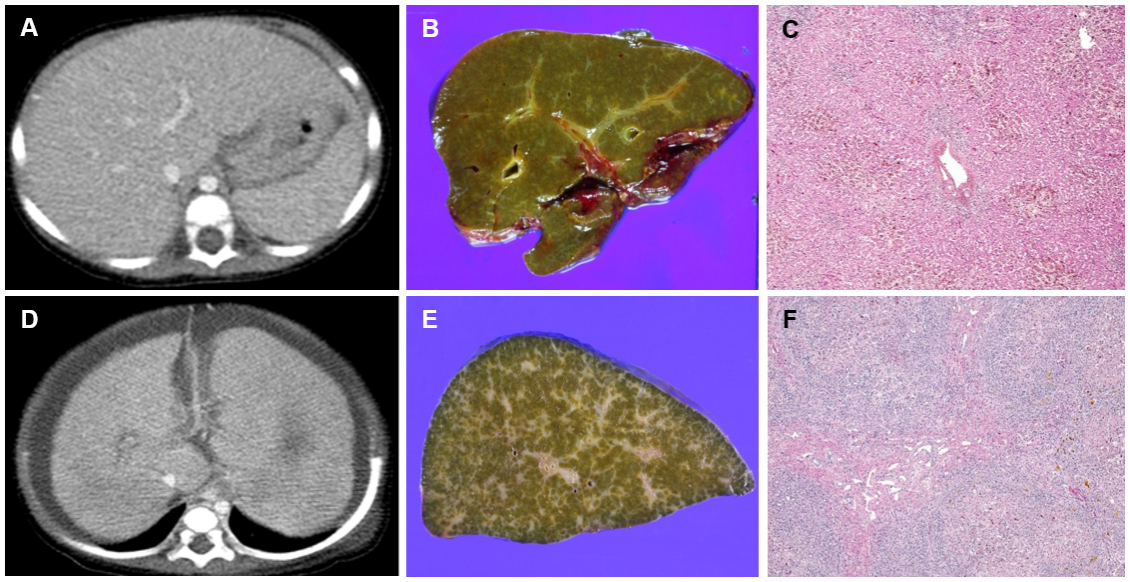
Contrast-enhanced axial CT scans for hepatic parenchymal changes. (A) Normal hepatic morphology in a 7-month-old girl with Alagille syndrome. (B, C) Macroscopic (B) and microscopic (C) specimens of the liver show cholestasis with paucity of interlobular bile duct, but parenchymal destruction is relatively mild (original magnification of microscopic image: x40). (D) Hepatic parenchymal changes with heterogeneous enhancement, fissure widening, left lobe hypertrophy, and periportal edema in a 5-month-old girl with biliary atresia. Note the presence of ascites and gastroesophageal varix. (E, F) Macroscopic (E) and microscopic (F) specimens of the liver show cholestasis and cirrhotic changes with marked parenchymal destruction (original magnification of microscopic image: x40).

The CT findings in AGS and BA patients are summarized in Table 3. Hepatic parenchymal changes were less common in AGS patients (3/11, 27%) than in BA patients (109/109, 100%) (*P*<0.001). Three patients (27%) with AGS exhibited hepatic parenchymal changes including heterogeneous parenchymal enhancement (n = 2) and periportal edema (n = 1). Whereas all patients with end-stage BA had hepatic parenchymal changes, with fissure widening being the most common (105/109, 96%) followed by heterogeneous enhancement (86/109, 79%), surface nodularity (84/109, 77%), periportal edema (79/109, 72%), and left lobe hypertrophy (30/109, 28%) (Fig 1).

**Table 3:** CT imaging findings in patients with Alagille syndrome and end-stage biliary atresia.

Variables	Alagille syndrome (n = 11)	End-stage biliary atresia (n = 109)	*P*-value
Hepatic parenchymal changes	3/11 (27%)	109/109 (100%)	<0.001
Heterogeneous enhancement	2/11 (18%)	86/109 (79%)	<0.001
Surface nodularity	0/11 (0%)	84/109 (77%)	<0.001
Fissure widening	0/11 (0%)	105/109 (96%)	<0.001
Left lobe hypertrophy	0/11 (0%)	30/109 (28%)	0.063
Periportal edema	1/11 (10%)	79/109 (72%)	<0.001
Vascular changes^a^			
Hepatic artery diameter (mm)	1.9 ± 0.6	3.6 ± 0.7	0.008
Portal vein diameter (mm)	6.8 ± 1.8	5.0 ± 1.6	<0.001
Ratio^b^	0.28 ± 0.06	0.77 ± 0.24	<0.001
Focal lesions	0/11 (0%)	48/109 (44%)	0.008
Intrahepatic biliary cysts	0/11 (0%)	43/109 (39%)	0.006
Hepatic tumors	0/11 (0%)	9/109 (8%)	0.597
Signs of portal hypertension	8/11 (73%)	109/109 (100%)	0.090
Splenomegaly	8/11 (73%)	105/109 (96%)	0.082
Ascites	1/11 (9%)	54/109 (50%)	0.010
Gastroesophageal varix	0/11 (0%)	87/109 (80%)	<0.001

Note.–Unless otherwise noted, data are numbers of patients with percentages in parentheses.

^a^Data are means ± standard deviation.

^b^Ratio means hepatic artery diameter to portal vein diameter

**Fig 2:**
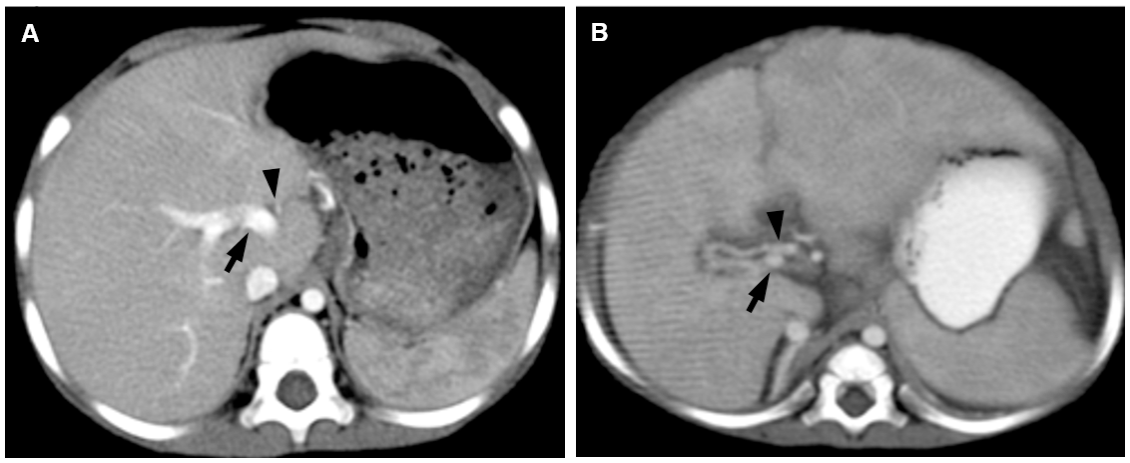
Contrast-enhanced axial CT scans for vascular changes. (A) Normal hepatic vasculature in a 5-month-old boy with Alagille syndrome. Diameters of proximal right hepatic artery (arrowhead) and portal vein (thin arrow) are 1.3 mm and 5.0 mm, respectively. The ratio of the hepatic artery diameter to the portal vein diameter is 0.26. (B) Enlarged hepatic artery and small portal vein diameters in a 6-month-old girl with biliary atresia. Diameters of proximal right hepatic artery (arrowhead) and portal vein (thin arrow) are 2.4 mm and 2.6 mm, respectively. The ratio of the hepatic artery diameter to the portal vein diameter is 0.92.

All of the AGS patients exhibited normal hepatic vasculature. The diameter of the hepatic artery in AGS patients (1.9 ± 0.6 mm) was significantly smaller than that in patients with end-stage BA (3.6 ± 0.7 mm) (*P* = 0.008), whereas the diameter of the portal vein in AGS patients (6.8 ± 1.8 mm) was significantly larger than that in patients with end-stage BA (5.0 ± 1.6 mm) (*P*<0.001). The ratio of the hepatic artery diameter to the portal vein diameter in the AGS patients (0.28 ± 0.06) was significantly larger than that in end-stage BA patients (0.77 ± 0.24) (*P*<0.001) (Fig 2).

No focal hepatic lesions were found in AGS patients, whereas patients with end-stage BA showed intrahepatic biliary cysts (43/109, 39%) and hepatic tumors (9/109, 8%) on CT scan. Intrahepatic biliary cysts were solitary and simple cysts (2/43, 5%) or multiple and complicated cysts (41/43, 95%). All hepatic tumors were identified as a solitary lesion and were confirmed as focal nodular hyperplasia (n = 5), regenerative nodule (n = 3), and cholangiocarcinoma (n = 1), based on histopathologic analysis of explanted liver. The tumors were observed as a well-defined (n = 7) or ill-defined (n = 2) mass with a mean size of 2.6 cm (range, 0.7–7 cm). Additionally, 2 hepatic tumors (one hepatocellular carcinoma [HCC], one regenerative nodule) were identified at the explanted liver, but these were not visible on the CT images.

The incidence of splenomegaly did not differ significantly between patients with AGS (8/11, 73%) and those with end-stage BA (105/109, 96%) (*P* = 0.082). However, the size of the spleen was significantly larger in patients with end-stage BA (11.2 ± 2.6 cm) compared with that in AGS patients (7.9 ± 3.0 cm) (*P* = 0.005). The presence of ascites (9% [1/11] versus 50% [54/109], *P* = 0.010) and gastroesophageal varix (0% [0/11] versus 80% [87/109], *P*<0.001) was less common in AGS patients than in end-stage BA patients.

## Discussion

Our study revealed that fibrotic or cirrhotic changes were less common in AGS than in end-stage BA, although both patients with AGS and those with end-stage BA showed cholestasis and abnormalities in biochemical hepatic function without statistically significant differences. Compared with end-stage BA, AGS is characterized by less common hepatic parenchymal changes, smaller portal vein and larger hepatic artery diameters, lack of focal lesions, and less common signs of portal hypertension at the pre-operative CT before liver transplantation.

Recent immunohistochemical study showed that epithelial components of the hepatic reparative mechanism are different between AGS and BA [9]. AGS is characterized by the near absence of reactive ductular cells and hepatic progenitor cells, which are significantly increased in BA. Because the reactive ductular cells and hepatic progenitor cells correlate positively with portal septa thickness, AGS shows less thickened portal septa than BA. Positive correlation between portal septal thickness and histologic staging of cirrhosis indicates that hepatic cirrhosis is less severe in AGS than in BA [14]. In addition, activation of different stem/progenitor cells regenerative compartments is recently suggested in the disease of liver, bile duct system and pancreas [15]. According to Carpino et al. [16], activation of peribiliary glands, which is niche of biliary tree stem cells, is triggered in primary sclerosing cholangitis—analogous with BA—affecting large intrahepatic and extrahepatic bile duct, whereas peribiliary glands hyperplasia is almost absent in primary biliary cirrhosis—analogous with AGS—affecting interlobular bile ducts. This difference in hepatic reparative mechanisms may explain the different imaging findings of hepatic parenchymal changes and the degree of portal hypertension between two cholestatic cholangiopathies of AGS and end-stage BA.

Another plausible explanation for different imaging findings of two cholestatic conditions is the indications for liver transplantation. Most children with end-stage BA undergo liver transplantation due to decompensation of synthetic function, uncontrolled portal hypertension, and recurrent cholangitis [17, 18]. However, in the majority of AGS patients, the indications for liver transplant comprise the complications of profound cholestasis and poor quality of life such as intractable pruritus, failure to thrive, hypercholesterolemia, and osteodystrophy [4, 6, 7] in concordance with our study.

Our study revealed that 8 patients (73%) with AGS showed no hepatic parenchymal changes, and the most common abnormality at CT imaging was splenomegaly, which was present in 73% (8/11) of patients with AGS. The reported radiologic appearances of AGS are diverse and previous studies demonstrated a much higher rate of hepatic parenchymal changes when compared with our data. Halvorsen et al [19] observed spherical hepatic deformity in 63% (5/8) of AGS patients. Berrocal et al [20] reported that 68% (19/28) of AGS patients showed hepatomegaly, fibrosis, or cirrhosis. The less common identification of hepatic parenchymal changes in our AGS patients could be explained by the patients’ age. AGS is progressive cholangiopathy, and the prevalence and severity of PIBD can increase with increasing age as a result of retained toxins, inflammation, or disuse atrophy. The age of patients in previous studies range from 3 months to 33 years [19, 20], and therefore included an older age group than in our study.

Previous radiologic study described displacement of portal vein or hepatic vein due to hepatic contour deformity in 50% (4/8) patients with AGS, but all of them showed normal hepatic vasculature [19], as seen in our series. In accordance with other studies based on radiologic, angiographic, and operative findings [21–25], our patients with BA showed enlarged hepatic artery and decreased portal vein diameters. These vascular alterations might be progressive and can be postulated by ongoing intrahepatic fibro-inflammatory process and cirrhosis [23–25]. Hepatic artery enlargement may be a compensatory change to improve the blood supply to the biliary systems [23, 24]. The diameter of portal vein would decrease in patients with end-stage BA before liver transplantation due to recurrent cholangitis and portal hypertension [22, 24, 25]. Hernandez-Cano et al [26]. noted that the portal vein diameter is reduced in BA patients with poor hepatobiliary function. As hepatic dysfunction is worsening and portal venous pressure is increasing, more portosystemic shunts would develop. The portosystemic shunts can divert blood flow from portal venous system and make the portal vein diameter decreasing [26–28].

Intrahepatic biliary cysts appeared only in patients with end-stage BA and in a considerable percentage (39%) of these patients, similar to previous reported incidences of 16%-42% [18, 25, 29]. Although the mechanism of intrahepatic biliary cysts formation after Kasai portoenterostomy is uncertain, fibro-obliterative and inflammatory process of the bile ducts have been proposed [18, 29].

Few cases of associated hepatic tumor such as HCC, hepatic nodular hyperplasia, or regenerative nodule in AGS have been reported. An association between HCC and biliary cirrhosis caused by AGS has been reported in 12 children in the literature [30–37]. Most (75%, 9/12) of these cases of HCCs developed in children aged 2 to 4 years [30–36], and all had histopathologically confirmed (83%, 10/12) [30–35, 37] or clinico-radiologically relevant (17%, 2/12) [34, 36] biliary cirrhosis. Hepatic tumors were identified in 10% of children with end-stage BA in this study, and all of cases were previously reported in the literature [38]. HCC is the most common malignant tumor in children with BA, and approximately half (56%, 15/27) of these HCCs occur in children younger than 4 years [38, 39]. Thus, we recommend that early surveillance for HCC should be performed by screening for serum alpha-fetoprotein and cross-sectional imaging in patients with AGS-related or BA-related cirrhotic liver.

To the best of our knowledge, this is one of the first studies to compare imaging findings between AGS and end-stage BA, although our study may be limited by small number of children with AGS and its retrospective nature. A large-scale investigation of AGS including a range of severity of phenotypic findings may be necessary.

## Conclusions

The histologic difference between AGS and BA may be reflected in preoperative CT findings before liver transplantation. Fibrotic or cirrhotic changes of the liver, presence of focal hepatic lesions, and relevant portal hypertension were less common in patients with AGS than in patients with end-stage BA.
